# Lupus acceleration by a MAVS-activating RNA virus requires endosomal TLR signaling and host genetic predisposition

**DOI:** 10.1371/journal.pone.0203118

**Published:** 2018-09-10

**Authors:** Rosana Gonzalez-Quintial, Anthony Nguyen, Dwight H. Kono, Michael B. A. Oldstone, Argyrios N. Theofilopoulos, Roberto Baccala

**Affiliations:** Department of Immunology and Microbiology, The Scripps Research Institute, La Jolla, California, United States of America; Universite Paris-Sud, FRANCE

## Abstract

Viruses have long been implicated in the pathogenesis of autoimmunity, yet their contribution remains circumstantial partly due to the lack of well-documented information on infections prior to autoimmune disease onset. Here, we used the lymphocytic choriomeningitis virus (LCMV) as a model to mechanistically dissect the impact of viral infection on lupus-like autoimmunity. Virus persistence strongly enhanced disease in mice with otherwise weak genetic predisposition but not in highly predisposed or non-autoimmune mice, indicating a synergistic interplay between genetic susceptibility and virus infection. Moreover, endosomal Toll-like receptors (TLRs) and plasmacytoid dendritic cells (pDCs) were both strictly required for disease acceleration, even though LCMV also induces strong TLR-independent type I interferon (IFN-I) production *via* RNA helicases and MAVS in conventional DCs. These results suggest that LCMV enhances systemic autoimmunity primarily by providing stimulatory nucleic acids for endosomal TLR engagement, whereas overstimulation of the MAVS-dependent cytosolic pathway in the absence of endosomal TLR signaling is insufficient for disease induction.

## Introduction

Systemic lupus erythematosus (SLE) is characterized by hyperactivation of T cells, B cells and DCs, production of autoantibodies to nucleic acid-associated and other self-molecules, and immune complex-mediated inflammatory damage in multiple organs [1]. Mechanistic assessments in both mouse models and humans have highlighted the critical role of innate immune pathways of nucleic acid sensing in lupus pathogenesis, including the engagement of endosomal Toll-like receptors (TLRs) in B cells and plasmacytoid dendritic cells (pDCs), production of type I interferons (IFN-I), and expression of an IFN-I inducible gene signature often correlating with disease activity [2–6]. In addition, evidence for the contribution of cytosolic RNA and DNA sensing has been presented [7–14], although whether overactivation of this pathway is sufficient to drive disease in the absence of endosomal TLR signaling remains to be determined.

Epidemiological and family studies have unequivocally established a strong genetic basis for SLE susceptibility [15]. Nevertheless, genetic predisposition is often insufficient, as documented by the incomplete concordance for SLE (~20–50%) in monozygotic twins [16–18]. Differences between genetically identical individuals may be due to stochastic events (e.g., T and B cell repertoire composition and epigenetic effects) and/or environmental contributions. Indeed, multiple environmental factors have been associated with lupus and other autoimmune conditions, with infectious agents receiving prominent attention [19].

The strongest association between infection and SLE has been reported for Epstein-Barr virus (EBV), a ubiquitous DNA virus that latently infects ~95% of the human population. EBV promotes B cell proliferation, and some of the encoded proteins, such as nuclear antigen 1 (EBVN-1), share sequence homology with SLE autoantigens and may induce *via* antigenic mimicry antibodies that crossreact with the ribonucleoproteins (RNPs) Ro, Sm B/B’ and Sm D1 [20]. Besides EBV, tentative associations with SLE have been reported for parvovirus B19 [21], cytomegalovirus [22], and polyomavirus [23].

These associations are suggestive and more studies are required to determine whether and how viral infections contribute to lupus pathogenesis. Early investigations found that chronic infection induced at birth with lymphocytic choriomeningitis virus (LCMV), a non-cytolytic negative-strand RNA virus [24], significantly enhanced disease in lupus-prone NZB, NZW, and NZB/W mice [25]. The current understanding of how viruses are sensed and provoke innate immune responses prompted us to examine mechanistically the impact of LCMV infection on autoimmune disease onset and progression. For this purpose, we used mice with various grades of systemic autoimmunity and found that persistent LCMV infection aggravated disease in genetically predisposed but not normal background mice. Moreover, the observation that LCMV-associated RNA engages both MyD88-dependent TLR7 in endolysosomes and MAVS-dependent MDA5 or RIG-I in the cytosol provided an experimental system to assess the differential contribution of these two pathways to disease enhancement. We found that lupus-predisposed mice lacking either endosomal TLR function or pDCs were protected from lupus-like disease even when persistently infected with LCMV. Thus, pDCs and endosomal TLRs are essential components of the core mechanism underlying pathogenic systemic autoimmunity, and their absence cannot be overcome by sustained stimulation of alternative innate immune cells and signaling pathways.

## Materials and methods

### Viruses

LCMV ARM and Cl13 stocks were prepared by serial passage in BHK-21 cells as described [26]. Resequencing and analysis of a specific *Mnl*I restriction site in the LCMV stocks [27] ensured the fidelity of the viruses used. Virus titers were determined by plaque assays on VeroE6 cells [28].

### Mice

NZB, BXSB, C57BL/6 and C57BL/6.*Mavs*^*–/–*^mice were purchased from the Jackson Laboratory (Bar Harbor, ME) or The Scripps Research Institute Animal Facility. Generation of C57BL/6.*3d* (*Unc93b1*^*3d/3d*^) mice [29], congenic NZB.*Irf8*^−/−^mice [30], and congenic NZB.*3d* mice [31] was described previously. The *Irf8*^−/−^ NZB mice and corresponding wild-type (WT) controls used in this study were littermates generated by crossing heterozygous *Irf8*^+/−^ mice. Homozygous *Unc93b1*^*3d/3d*^*Mavs*^*–/–*^(*3d/Mavs*) double-deficient C57BL/6 mice lacking both endosomal TLR and MAVS signaling were generated by crossing C57BL/6.*3d* and C57BL/6.*Mavs*^*–/–*^mice. Similar double-deficient mutants in mixed (NZB×C57BL/6) background were generated as (NZB.*3d*×C57BL/6.*3d/Mavs*)×C57BL/6.*3d/Mavs* backcrosses, followed by genotyping to select for *3d/Mavs* homozygosity. Estimation of group sizes was based on previous experience with LCMV-infected and lupus-prone mice without a priori determination by power calculation. Individual mice were randomly assigned to experimental groups and analyzed under identical experimental conditions but without blinding except for GN score determinations. Neonatal infection with Cl13 or ARM was performed by injection of 10^3^ plaque-forming units (PFU) per mouse. Efficiency of infection was verified by measuring serum virus titers by plaque assay in adult (2–3 mo old) individual mice, which showed titers ranging from 0.6 × 10^5^ to 1.4 × 10^5^ PFU/mL, with no significant differences between male and female mice or among mouse strains. For serum analysis, blood was drawn by retro-orbital puncture under anesthesia (isoflurane), and serum was isolated by low speed centrifugation and frozen until use. Except in rare cases when mice were found dead (<20% of the mice), throughout this study we used humane endpoint criteria including in experiments determining survival/mortality rates, i.e. mice were monitored daily and humanely euthanized under anesthesia (isoflurane) when exhibiting any of the following symptoms for longer than 24 hrs, including hunched posture with lethargy, labored breathing, lack of righting reflex, no response to mild stimuli, and signs of hind area edema.

### Study approval

Mice were housed under specific pathogen-free conditions, and all experimental protocols were performed according to the NIH Guide for the Care and Use of Laboratory Animals and approved by The Scripps Research Institute Animal Care and Use Committee.

### Cell preparations

Single cell suspensions were prepared from bone marrow (BM), blood, and spleen. B cells were purified from spleen using magnetic beads (MACS, Miltenyi Biotec), while enriched populations of pDCs were prepared by culturing BM cells with recombinant FMS-like tyrosine kinase 3 ligand (Flt3L, R&D Systems) [30, 32].

### FACS

For surface staining, cells were incubated with fluorochrome- or biotin-labeled antibodies specific for mouse CD11c, PDCA-1, Siglec-H, B220, CD11b, CD4, CD8, CD44, CD19, CD21, CD23, CD86, I-A^d^ or H2-K^d^ (BD Pharmingen, Biolegend or eBioscience), followed by streptavidin in the case of biotinylated antibodies, as described [30, 32]. Cells were then washed with PBS and fixed with 4% paraformadehyde. For intracellular staining, fixed cells were permeabilized with 2% saponin or permeabilization solution (BD, Pharmingen), and incubated with specific antibodies (anti-Foxp3; anti-LCMV-NP, VL-4). Surface and intracellularly stained cells were acquired on a BD FACSDiva-driven LSR II flow cytometer, and data were analyzed using FlowJo software (Treestar).

### ELISA

Serum levels of polyclonal IgM and IgG and anti-chromatin IgG subclasses were determined in individual mice by triplicate ELISA analysis using 96-well plates coated with goat anti-mouse immunoglobulin (Jackson ImmunoResearch Laboratories) or chromatin, respectively. Bound antibodies were detected using alkaline phosphatase-conjugated goat antibodies to mouse IgM, IgG or IgG subclasses (Southern Biotech), and standard curves were generated using calibrated mouse serum (Nordic Immunology). Commercial ELISA kits were used to examine IFN-α (PBL InterferonSource), TNF-α or IL-6 (Biolegend) levels in mouse sera and supernatants of B cell and DC cultures.

### Luminescence bioassay

Serum levels of IFN-I induced by LCMV infection were determined using a mouse L929 cell line stably transfected with a IFN-stimulated response element-dependent luciferase (ISRE-luc) construct. These cells were incubated with mouse serum for 5 h, washed, lysed with reporter lysis buffer (Promega), and frozen overnight at -80 °C. After thawing, luciferase solution was added, and luminescence was read 5 min later.

### Anti-nuclear and anti-erythrocyte autoantibodies

Anti-nuclear autoantibodies (ANA) were detected by incubating HEp-2 slides (Bion Enterprises) with individual mouse serum (serial dilutions of 1/10 to 1/3000), followed by Alexa-Fluor 488-conjugated goat anti-mouse IgG (Invitrogen). Fluorescence intensity was quantified for each mouse using ImageJ (National Institute of Health). Erythrocyte-bound autoantibodies were quantified by flow cytometry after sequential incubation of washed red blood cells (RBC) with biotinylated goat anti-mouse IgG (MP Biomedicals) and streptavidin-PE (Biolegend). For each sample, the geometric mean fluorescence intensity (GMFI) was calculated by subtracting the background determined by incubating washed RBCs with streptavidin-PE only.

### *In vitro* and *in vivo* TLR responses

Purified splenic B cells and BM-derived cDCs and pDCs isolated from individual mice were stimulated or not with mouse IFN-α11 (1000 U/ml, Miltenyi Biotec), the TLR7 ligand R848 (1 μg/ml, InvivoGen), the TLR9 ligand ODN-2216 (CpG, 1 μg/ml, InvivoGen), anti-IgM (20 μg/ml, eBioscience), or combinations thereof. At the indicated time-points, cells were harvested, counted, and analyzed by flow cytometry, while supernatants were assayed for cytokines by ELISA. *In vivo* TLR9 stimulation was induced by injecting mice i.v. with 2 μg CpG-A (ODN-2216) mixed with the cationic lipid DOTAP. Six hours later, serum was collected and tested for the presence of IFN-α by ELISA.

### Kidney pathology and immunohistology

Proteinuria was determined using Albustix strips (Bayer Corporation) and graded semiquantitatively (0 = negative to traces, 1 = 30mg/ml; 2 = 100 mg/ml, 3 = 300 mg/ml, 4 = 2000 mg/ml). Tissue sections were fixed in zinc formalin and stained with periodic acid-Schiff (PAS) or snap-frozen in OCT for immunofluorescence. Severity of glomerulonephritis (GN) was determined as described [33] by analyzing >50 representative glomeruli which were graded blindly on a 0 to 4 scale, where 0 = no pathology, 1 = minimal mesangial thickening, 2 = noticeable increases in both mesangium and glomerular cellularity, 3 = the preceding features plus inflammatory exudates and/or capsular adhesion, and 4 = obliteration of glomerular architecture involving >70% of glomeruli; a score ≥ 2.0 was considered pathologic. Kidney sections were also examined by immunofluorescence for the presence of immune deposits using antibodies to mouse IgG2a (Invitrogen) and C3 (Nordic Immunology). Fluorescence intensity was quantified for each mouse using ImageJ (National Institute of Health).

### Statistical analysis

Group comparisons were performed by unpaired two-tailed Student’s *t*-test. Survival was analyzed by Kaplan-Meier plot and log-rank test. *p* < 0.05 was considered significant.

## Results

### LCMV infection accelerates lupus-like disease in NZB mice

**Fig 1:**
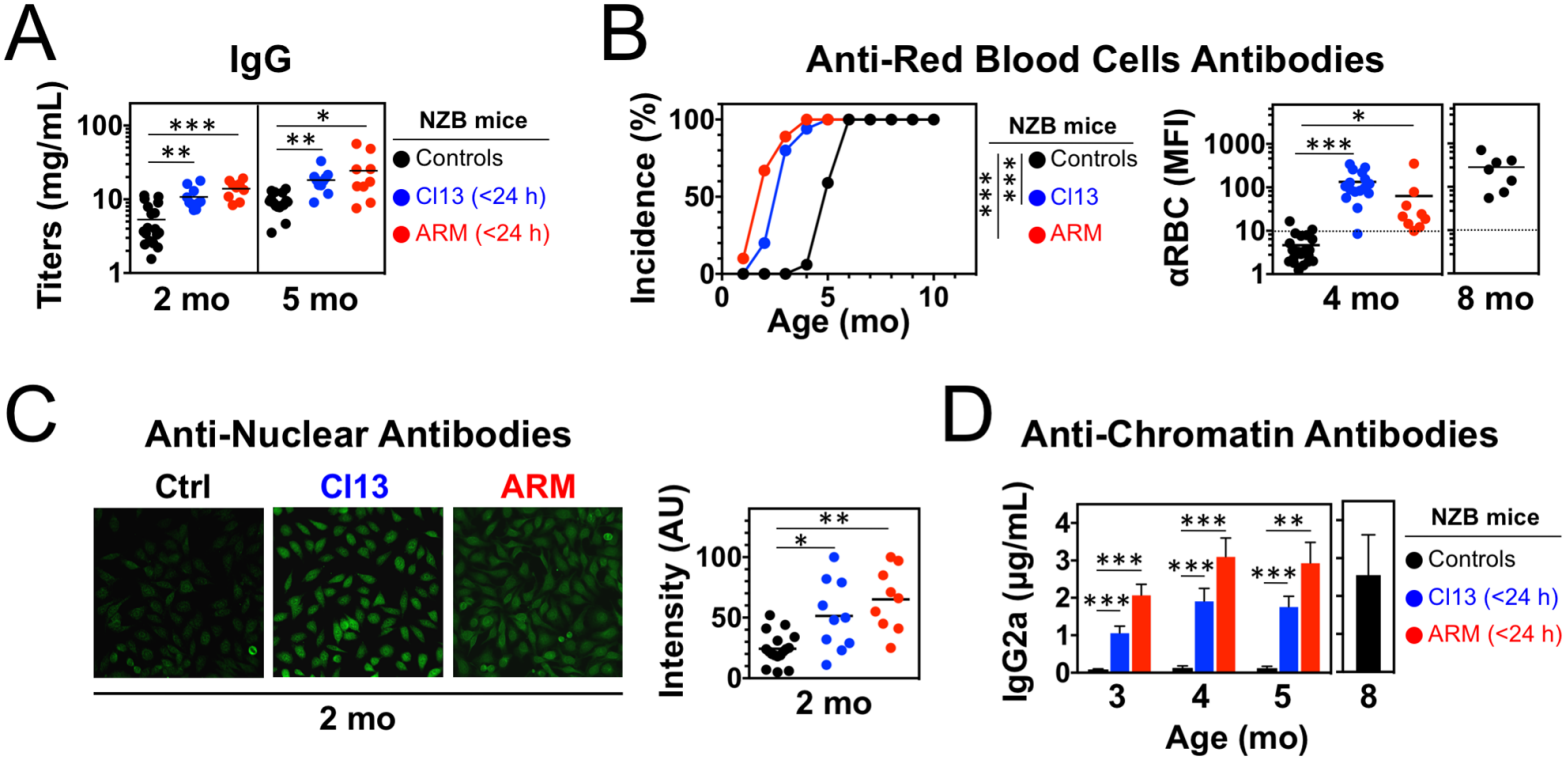
Increased autoantibody production in NZB mice neonatally infected with LCMV. Mice were infected <24 h after birth with LCMV Arm or Cl13 and analyzed at the indicated ages. Uninfected mice were used as controls. (**A**) Total serum IgG titers detected by ELISA (*n* = 9–18 mice). (**B**) Incidence and levels of red blood cell (RBC)-bound autoantibodies determined by FACS (*n* = 7–18 mice). (**C**) Anti-nuclear autoantibodies assessed by incubating mouse serum with HEp-2 cells and immunofluorescence analysis (*n* = 9–18 mice). (**D**) Anti-chromatin IgG2a autoantibodies detected by ELISA (sensitivity 5–10 ng/mL, *n* = 5–9 mice). Graphs summarize data from 2–3 independent experiments. Dots (in A-C) represent individual mice, horizontal bars (in A-C) indicate average, error bars (in D) indicate standard deviation, and asterisks indicate statistical significance (*, p<0.05; **, p<0.01; ***, p<0.001).

**Fig 2:**
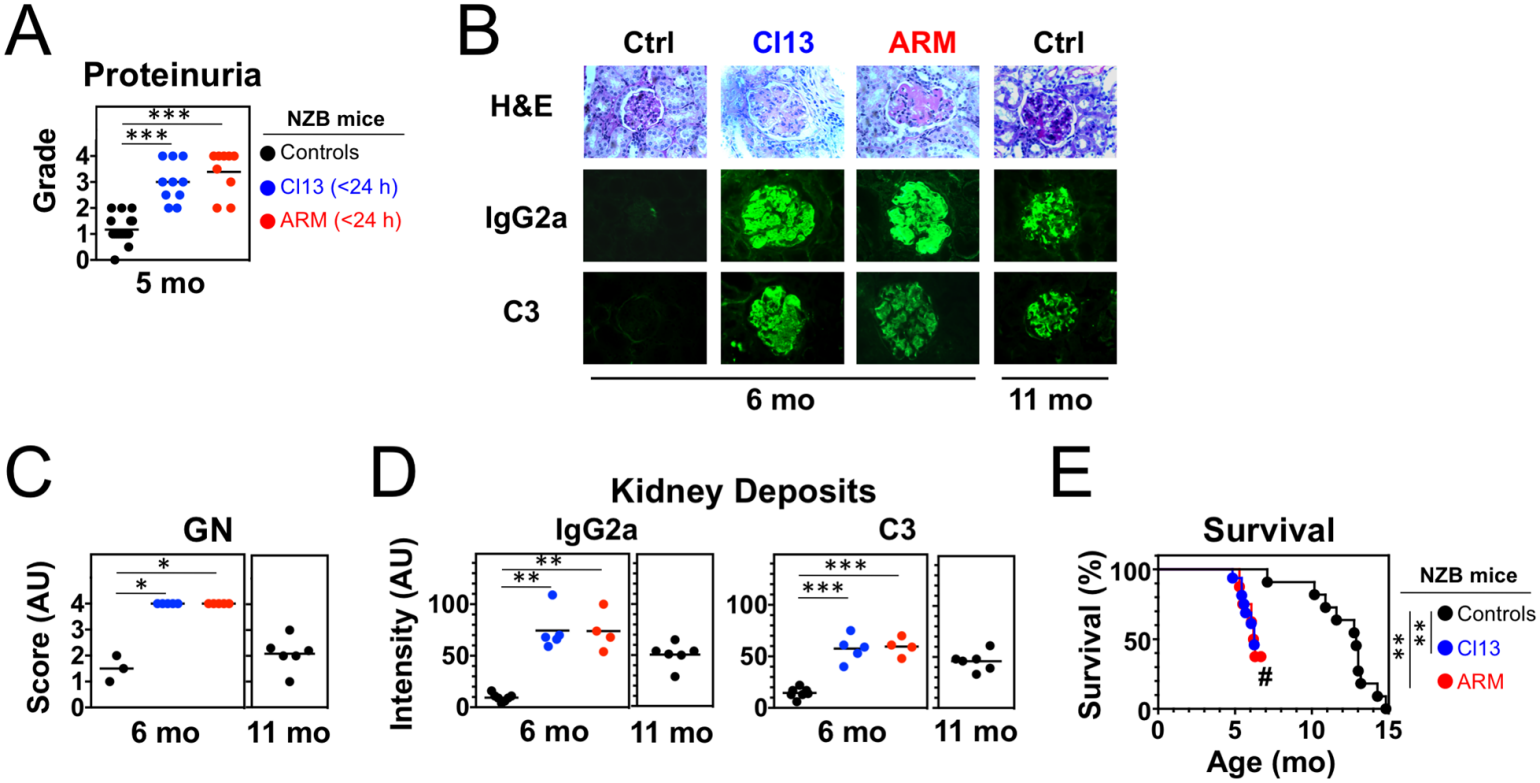
Enhanced kidney disease and mortality in NZB mice neonatally infected with LCMV. Mice were infected <24 h after birth with LCMV Arm or Cl13 and analyzed at the indicated ages. Uninfected mice were used as controls. (**A**) Proteinuria (*n* = 9–18 mice). (**B**) Kidney pathology with representative periodic acid-Schiff (PAS)-stained sections and immunofluorescence analysis of IgG2a and C3 deposits (*n* = 4–7 mice). (**C**) Glomerulonephritis scores (*n* = 3–6 mice). (**D**) Quantitation of glomerular IgG2a and C3 deposits defined by immunofluorescence (*n* = 4–7 mice). (**E**) Survival. (*n* = 8–16 mice; #, remaining mice were sacrificed when 50% mortality was attained). Plots summarize data from 1–2 independent experiments. Dots represent individual mice, horizontal bars indicate average, and asterisks statistical significance (*, p<0.05; **, p<0.01; ***, p<0.001).

To study the contribution of virus infections to lupus pathogenesis, we examined disease development in NZB mice persistently infected with two different LCMV isolates, Armstrong (ARM) and clone 13 (Cl13). When inoculated intravenously (i.v.) into adult mice of most strains, ARM induces the rapid generation of specific cytotoxic T lymphocytes (CTLs) that mediate virus clearance within 7–10 days, whereas Cl13 induces functional T cell exhaustion, resulting in a chronic infection typically lasting >90 days [24]. In contrast, either of these LCMV strains causes lifelong persistent infection in mice inoculated during fetal life or early after birth due to deletion or suppression of LCMV-specific T cells [34]. Accordingly, neonatal inoculation (<24 h after birth) of NZB mice with either ARM or Cl13 resulted in persistent infection, shown by serum virus titers of 1.01 ± 0.19 × 10^5^ PFU/mL in adult (2–3 mo-old) mice. We found that, at the age of 2 and 5 mo, infected NZB mice had significantly elevated serum polyclonal IgM (not shown) and IgG levels (Fig 1A). Similarly, IgG anti-erythrocyte autoantibodies, a typical manifestation of the NZB disease, were already detectable at 2 mo of age and reached high titers in >80% of the infected mice at 4 mo of age, when only <10% of uninfected controls were positive with low titers (Fig 1B). Infected NZB mice also exhibited increased levels of circulating IgG anti-nuclear autoantibodies (ANA) at age 2 mo (Fig 1C), while anti-chromatin IgG2a autoantibodies, the dominant subclass in this model, were detectable at 3 mo, i.e. ~3–4 mo earlier than in controls (Fig 1D). The high levels of autoantibodies in infected mice was associated with markedly accelerated renal disease, evidenced by increases in proteinuria (Fig 2A), IgG2a and C3 kidney deposits, and glomerulonephritis (GN) scores (Fig 2B–2D). Remarkably, 6 mo-old infected mice had worse GN scores than 11 mo-old uninfected controls. Consequently, survival was significantly shortened in infected mice, with a 50% mortality of 6 mo *vs*. 13 mo in the control group (Fig 2E). For all tested parameters, disease aggravation by LCMV infection was similar in male and female mice. These results show that persistent infection with LCMV severely accelerates lupus-like disease in NZB mice.

### Synergism between virus infection and genetic susceptibility

**Fig 3:**
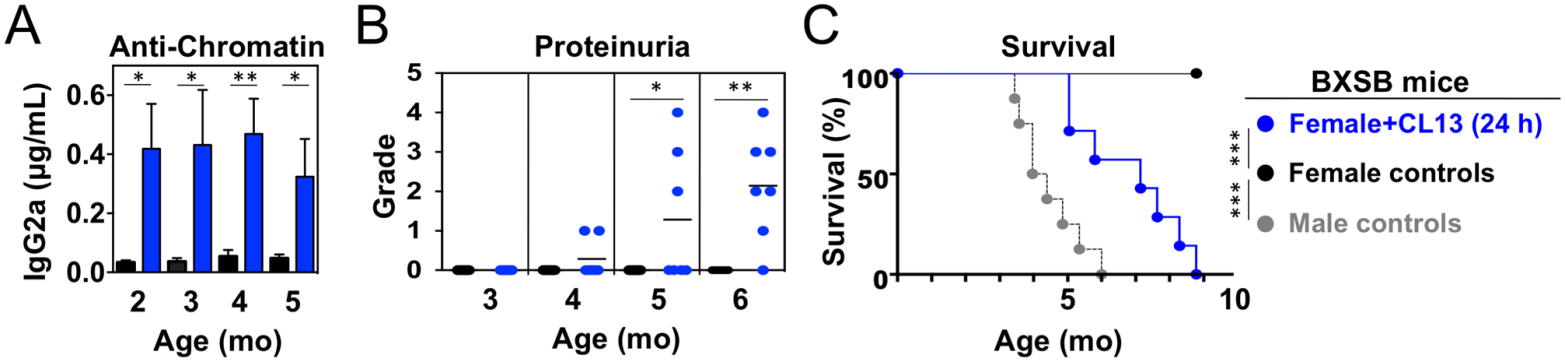
Neonatal LCMV infection aggravates lupus-like disease in female BXSB mice. Mice were infected <24 h after birth with LCMV Cl13 and analyzed at the indicated ages. Uninfected mice were used as controls. (**A**) Anti-chromatin IgG2a autoantibodies detected by ELISA (sensitivity 5–10 ng/mL, *n* = 5–7 mice). (**B**) Proteinuria (*n* = 5–7 mice). (**C**) Survival (*n* = 5–8 mice). Error bars (in A) indicate standard deviation, dots (in B) represent individual mice, horizontal bars (in B) indicate average, and asterisks indicate statistical significance (*, p<0.05; **, p<0.01; ***, p<0.001).

We next assessed the impact of neonatal viral infection on lupus-like manifestations in mice with various degrees of genetic susceptibility, including male BXSB mice with early-onset severe autoimmunity due to a Y chromosome-associated *Tlr7* gene duplication [35, 36], female BXSB mice with late onset, low-incidence mild disease, and non-autoimmune C57BL/6 mice. Persistent infection with LCMV Cl13 did not significantly accelerate the already severe disease of male BXSB mice, nor it induced autoimmunity in male or female C57BL/6 mice, based on anti-chromatin autoantibody levels, proteinuria, and mortality (S1 Fig). In contrast, disease was strongly accelerated in neonatally infected female BXSB mice, evidenced by earlier detection of IgG2a anti-chromatin autoantibodies (2 mo *vs*. >5 mo in controls), proteinuria (4–5 mo *vs*. >8 mo in controls), and 50% mortality (7.3 mo *vs*. >18 mo in controls) (Fig 3). Differences in disease among groups could not be attributed to variations in virus persistence, as indicated by similar serum titers in 2–3 mo old mice (1.04 ± 0.32 × 10^5^ PFU/mL in male BXSB; 0.95 ± 0.15 × 10^5^ in female BXSB; 0.93 ± 0.08 × 10^5^ in male C57BL/6 mice; 0.96 ± 0.22 × 10^5^ in female C57BL/6 mice). Thus, neonatal LCMV infection can, to a significant extent, mimic the effect of the *Tlr7* duplication, leading to enhanced disease severity in female BXSB mice with otherwise weak genetic predisposition.

### Enhanced accumulation of activated B cells, T cells and pDCs in persistently infected lupus mice

To define the mechanistic basis of virus-induced autoimmunity acceleration, we assessed the effect of infection on the cellularity and activation phenotype of innate and adaptive immunocyte populations. Consistent with earlier findings in non-autoimmune C57BL/6 mice [37] and reflecting the cellular distribution of alpha-dystroglycan, the main receptor for LCMV [24, 38], neonatal inoculation of NZB mice with LCMV Cl13 resulted in the persistent infection of ~3–4% of spleen cells. Various degrees of infectivity were noted depending on cell type (S2 Fig), with minimal infection rates of B and T cells, except marginal zone B cells (9% LCMV^+^), and high infection rates of pDCs (45%), cDCs (49%), macrophages (81%), as well as monocytes and other CD11b^+^ cells (19%), of which Gr-1^int^ cells were the most infected (38%), followed by Gr-1^hi^ cells (14%) and Gr-1^neg^ cells (11%).

**Fig 4:**
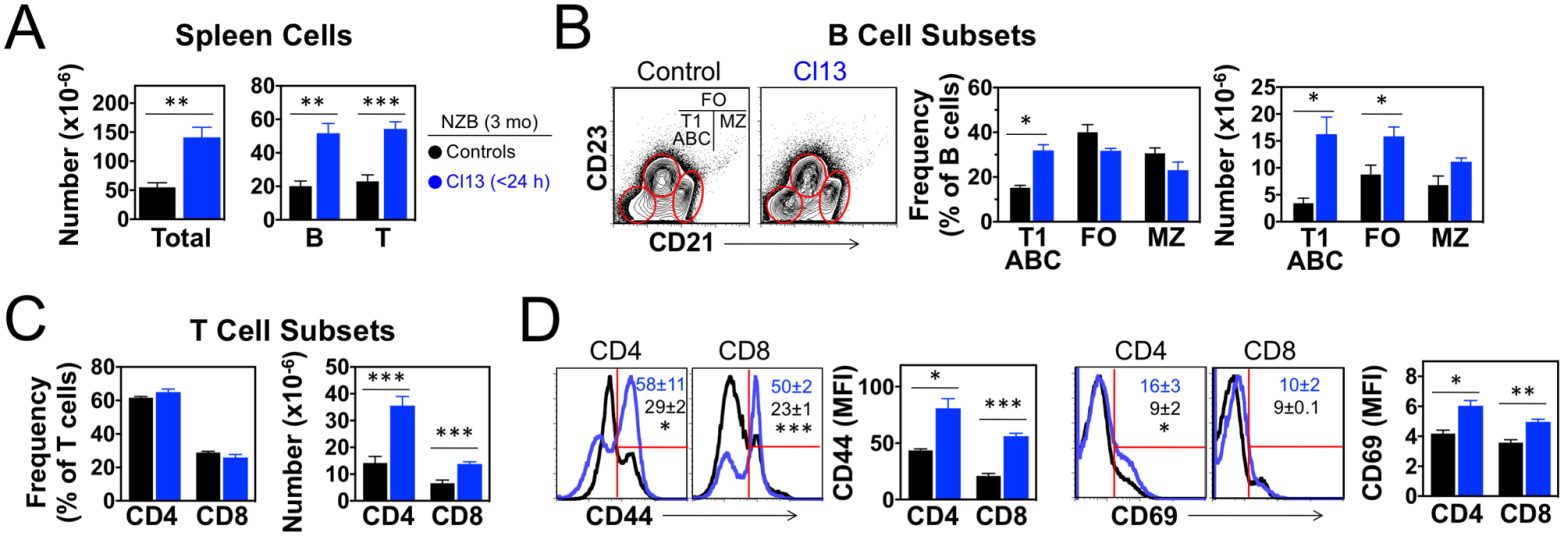
Expansion of activated B and T cells in NZB mice neonatally infected with LCMV. Mice were infected <24 h after birth with LCMV Cl13 and spleen cells analyzed at the age of 3 mo. Uninfected mice were used as controls. (**A**) Number of total cells, B cells and T cells in spleen (*n* = 6 mice). (**B**) FACS analysis of CD21^−^CD23^−^ (T1 B cells and ABCs), CD21^low^CD23^+^ (follicular B cells, FO), and CD21^+^CD23^−^ (marginal zone B cells, MZ) B cell subsets within gated IgM^+^B220^+^ splenocytes (*n* = 3 mice). (**C**) Frequency and numbers of CD4^+^ and CD8^+^ T cells identified by FACS in gated live spleen cells (*n* = 6 mice). (**D**) T cell activation status defined by FACS analysis of CD44 and CD69 surface levels (expressed as mean fluorescence intensity, MFI) in gated CD4^+^ and CD8^+^ T cells (*n* = 3 mice). Numbers within FACS histograms indicate percent (± standard deviation) of CD44^hi^ and CD69^+^ cells in the spleen of Cl13-infected (blue) or uninfected (black) mice. Data are representative of 2 independent experiments. Error bars indicate standard deviation and asterisks statistical significance (*, p<0.05; **, p<0.01; ***, p<0.001).

Disease enhancement in infected NZB mice was associated with significant increases in spleen cellularity (~2.5-fold in 3 mo-old mice) primarily attributable to the expansion of B and T cells (Fig 4A). Among B cell subsets, the expansion mostly affected the CD21^−^CD23^−^ population (Fig 4B), including the so-called “age-associated” B cells (ABCs), known to accumulate in aging humans and in mice and patients with systemic autoimmunity [39, 40]. The expansion of ABCs in infected NZB mice with accelerated disease was confirmed by analysis of CD19^+^CD11b^+^ cells at the age of 6 mo (18.7 ± 8.9% of CD19^+^ cells in infected mice *vs*. 7.4 ± 2.1% in non-infected controls, p<0.05). The number of CD21^low^CD23^+^ follicular B cells was also increased in 3 mo-old infected mice, whereas no significant changes were noted for CD21^+^CD23^−^ marginal zone B cells (Fig 4B). Expansion of the T cell compartment in infected NZB mice included both CD4^+^ and CD8^+^ subsets, which maintained approximately the same ratio as in uninfected controls (Fig 4C). CD4^+^CD25^+^Foxp3^+^ regulatory T cells [41] also increased proportionally in infected mice, resulting in a similar frequency as in control mice (24.2 ± 3.7% of CD4^+^ T cells in infected mice *vs*. 19.0 ± 2.8% in controls, p>0.05). Moreover, the enlarged T cell populations were significantly more activated in infected mice, shown by the increased expression of CD44 and CD69 and the increased frequency of cells displaying high levels of CD44 in CD4^+^ and CD8^+^ subsets, and CD69 in the CD4^+^ subset (Fig 4D).

**Fig 5:**
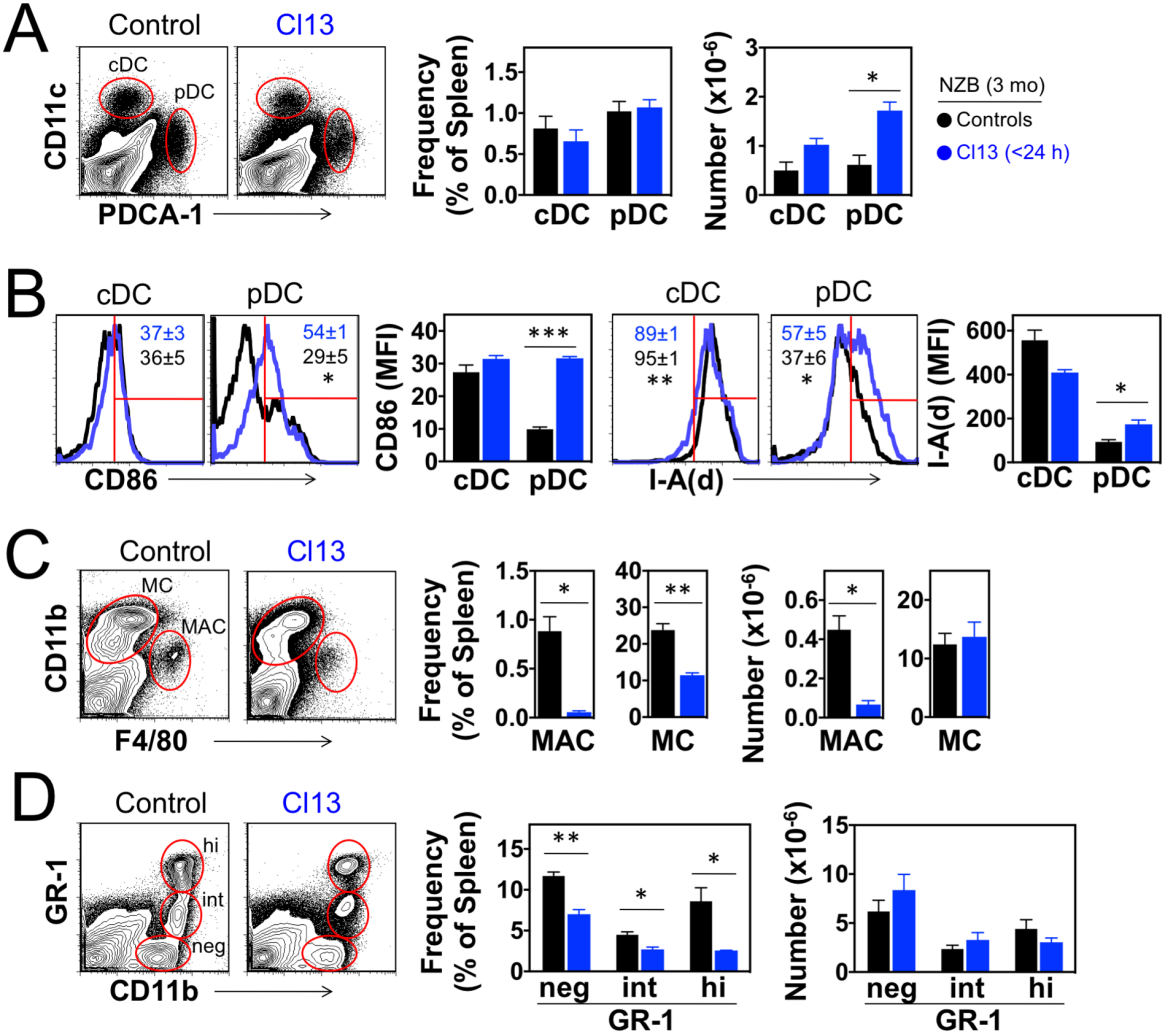
Expansion of activated pDCs in NZB mice neonatally infected with LCMV. Mice were infected <24 h after birth with LCMV Cl13 and spleen cells analyzed at the age of 3 mo. Uninfected mice were used as controls. (**A**) Frequency and number of cDCs (CD11c^+^ PDCA-1^−^) and pDCs (CD11c^low^ PDCA-1^+^) identified by FACS in gated live spleen cells (*n* = 3 mice). (**B**) Activation status of cDCs and pDCs indicated by expression levels of CD86 and MHC class II (I-A^d^) molecules measured by FACS as mean fluorescence intensity (MFI). Numbers within FACS histograms indicate percent (± standard deviation) of CD86^+^ and I-A(d)^hi^ cells in spleen of Cl13-infected (blue) or uninfected (black) mice (*n* = 3 mice). (**C**) Frequency and number of macrophages (CD11c^−^ CD11b^low^ F4/80^+^) and monocytes (CD11c^−^ CD11b^+^ F4/80^−^) identified by FACS in gated live spleen cells (*n* = 3 mice). (**D**) Frequency and number of CD11b^+^ monocyte subsets defined by Gr-1 marker expression in gated CD11c^−^ F4/80^−^ spleen cells (*n* = 3 mice). Data are representative of 2 independent experiments. Error bars indicate standard deviation and asterisks statistical significance (*, p<0.05; **, p<0.01; ***, p<0.001).

Analysis of innate immune cells showed a significant increase in the number of pDCs in the spleen of neonatally infected NZB mice compared to controls (Fig 5A). A trend toward increased cellularity was also noted for cDCs, although the difference did not reach statistical significance (Fig 5A). In infected mice, pDCs (but not cDCs) were also more activated, shown by upregulation of CD86 and MHC class II (I-A^d^) molecules (Fig 5B). Further analysis revealed that in infected mice LCMV^+^ pDCs expressed significantly higher levels of CD86 than LCMV^−^pDCs (Panel A in S3 Fig), suggesting activation by cell-intrinsic virus-sensing pathways. Nevertheless, LCMV^−^pDCs from infected mice displayed comparable levels of MHC class II as LCMV^+^ pDCs (Panel B in S3 Fig), suggesting additional contributions by cell-extrinsic factors, including IFN-I and other inflammatory cytokines produced in response to virus sensing. Likewise, in infected mice CD86 expression was slightly increased in LCMV^+^ relative to LCMV^−^cDCs (Panel C in S3 Fig), whereas MHC class II expression was rather decreased (Panel D in S3 Fig). Additional infection-associated variations included reductions in the frequency and numbers of macrophages (Fig 5C) and in the frequency of CD11b^+^ monocytes (Fig 5C and 5D), including Gr-1^neg^ cells known to expand in other models of spontaneous lupus [42]. Collectively, these results indicate that disease acceleration following neonatal inoculation of NZB mice with LCMV correlates with the persistent infection of large fractions of pDCs, cDCs, monocytes and macrophages, and accumulation of activated pDCs, B cells and T cells.

### LCMV infection potentiates B cell responses but reduces pDC responses to endosomal TLR stimulation

**Fig 6:**
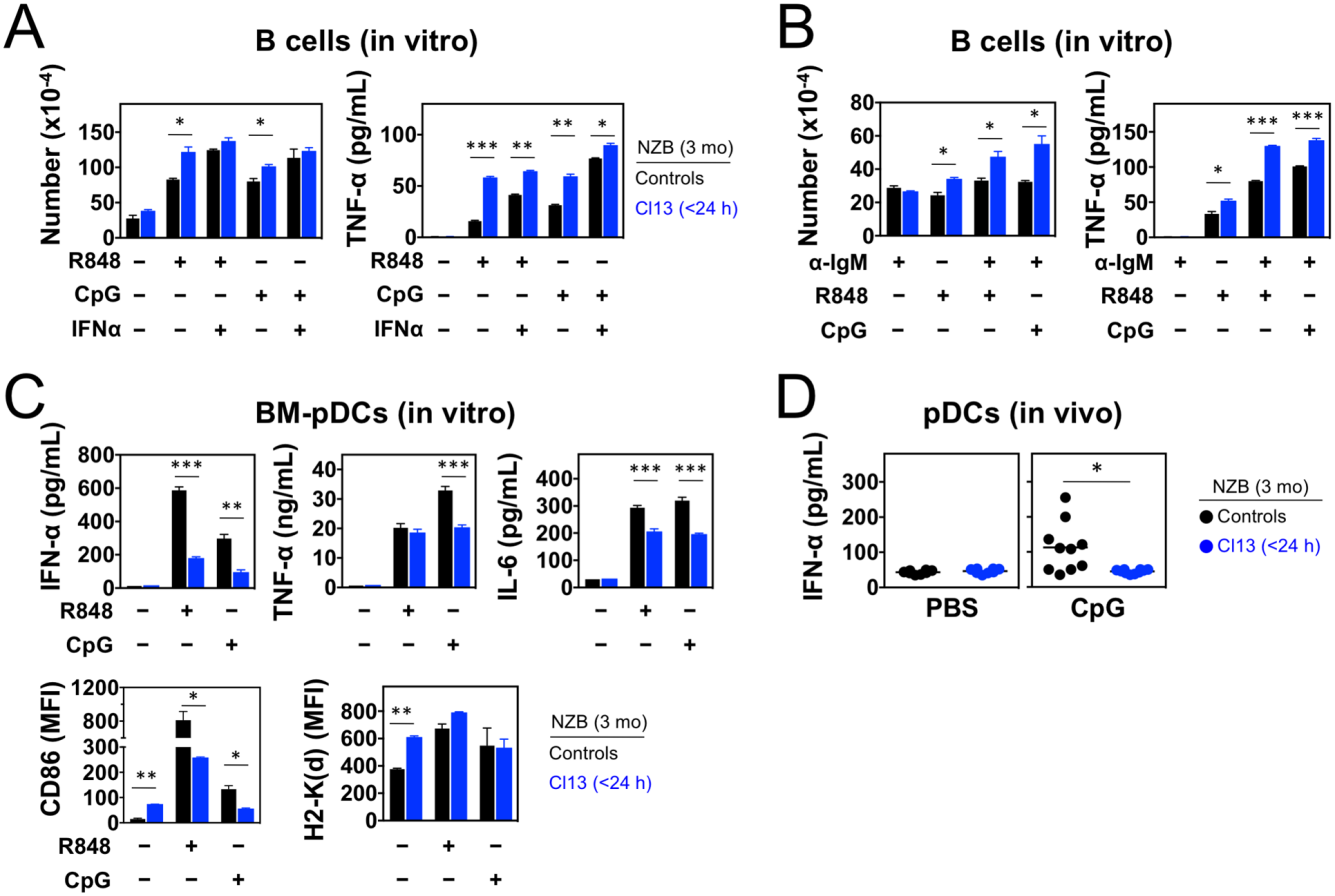
Enhanced B cell responses but reduced pDC responses to endosomal TLR stimulation in NZB mice neonatally infected with LCMV. Mice were infected <24 h after birth with LCMV Cl13 and analyzed at the age of 3 mo. Uninfected mice were used as controls. (**A**) Purified spleen B cells were stimulated *in vitro* with endosomal TLR ligands (R848 for TLR7, CpG for TLR9) in presence or absence of IFN-α. After 120 h, B cells were enumerated, and TNF-α levels in supernatants were assessed by ELISA (sensitivity 10–20 pg/mL, *n* = 2 mice). (**B**) Purified spleen B cells were stimulated *in vitro* with the indicated combinations of anti-IgM, R848 (TLR7 ligand) and CpG (TLR9 ligand). After 120 h, B cells were enumerated, and TNF-α levels in supernatants were assessed by ELISA (sensitivity 10–20 pg/mL, *n* = 2 mice). (**C**) BM-derived pDCs were stimulated *in vitro* (24 h) with TLR7 or TLR9 ligands. Levels of IFN-I, IL-6 and TNF-α in supernatants were assessed by ELISA (sensitivity 10–20 pg/mL), and cell surface expression of CD86 and MHC class I (H2-K^d^) were determined by FACS in gated PDCA-1^+^ B220^+^ cells (*n* = 2–4 mice). (**D**) Neonatally infected and control mice were injected with CpG ODN-2216 (TLR9 ligand) or PBS (control), and serum levels of IFN-α were assessed by ELISA (sensitivity 10–20 pg/mL) 6 h post-injection (*n* = 8–10 mice). Data are representative of 2–3 independent experiments. Error bars (in A-C) indicate standard deviation, horizontal bars (in D) indicate average, and asterisks statistical significance (*, p<0.05; **, p<0.01; ***, p<0.001).

A critical pathogenic event in spontaneous lupus is the engagement of endosomal TLRs by self-nucleic acids in B cells and pDCs, and this response could be magnified in LCMV-infected mice with accelerated disease. To address this possibility, purified splenic B cells from 3 mo-old NZB mice were stimulated *in vitro* with ligands for TLR7 (R848) or TLR9 (CpG DNA), in the presence or absence of IFN-α. B cells from infected mice responded more efficiently to TLR stimulation (Fig 6A), an effect possibly due to *in vivo* priming by virus-induced IFN-I since hyperresponsiveness was recapitulated with control B cells upon exposure to high concentration of IFN-α. Moreover, B cells from infected mice showed markedly increased responses to concurrent anti-IgM-mediated BCR crosslinking and TLR7 or TLR9 stimulation (Fig 6B).

We next compared TLR responses by pDCs from neonatally infected *vs*. control NZB mice. These cells were enriched by Flt3L-induced *in vitro* differentiation of bone marrow (BM) cells from 3 mo old donors. Differentiation was similarly efficient for cells from infected and control mice (Panel A in S4 Fig), even though approximately 51% of BM-pDCs from infected mice were LCMV^+^ (Panel B in S4 Fig), a rate comparable to that observed by *ex vivo* analysis of splenic pDCs (S2 Fig). Phenotypic characterization before TLR stimulation showed that BM-pDCs from infected mice were significantly more activated than those from control mice, indicated by upregulation of CD86 (Panel C in S4 Fig) and MHC class I (H2-K^d^) molecules (Panel D in S4 Fig). As observed with splenic pDCs, LCMV^+^ BM-pDCs were more activated and expressed higher levels of CD86 and H2-K^d^ than LCMV^−^BM-pDCs, with the latter displaying an activation profile very similar to BM-pDCs from uninfected control mice (Panels C and D in S4 Fig). Surprisingly, unlike B cells, BM-pDCs from infected mice responded less efficiently to TLR7 or TLR9 stimulation than BM-pDCs from uninfected mice, indicated by reduced secretion of IFN-I, TNF-α, and IL-6 and impaired upregulation of CD86 and H2-K^d^ (Fig 6C).

To corroborate *in vivo* the evidence that pDCs of infected mice exhibit defective endosomal TLR responses, neonatally infected and control NZB mice were injected intravenously at age 3 mo with the TLR9 agonist CpG-2216. Previous studies with knockin *Ifna6-GFP* [43] or *Ifnb-EYFP* [44] reporter mice showed that *in vivo* stimulation with CpG specifically activates IFN-I production by pDCs but not other cells, which was confirmed by the absence of serum IFN-I in pDC-deficient NZB.*Irf8*^*–/–*^mice injected with CpG-2216 (S5 Fig). Consistent with the *in vitro* results presented above, we found that infected mice failed to produce detectable amounts of IFN-I following *in vivo* CpG challenge (Fig 6D). Overall, these *in vitro* and *in vivo* findings indicate that, in persistently infected mice with accelerated lupus, B cells are hyperresponsive, whereas pDCs are hyporesponsive, to endosomal TLR stimulation.

### pDCs are required for LCMV-induced lupus acceleration in NZB mice

**Fig 7:**
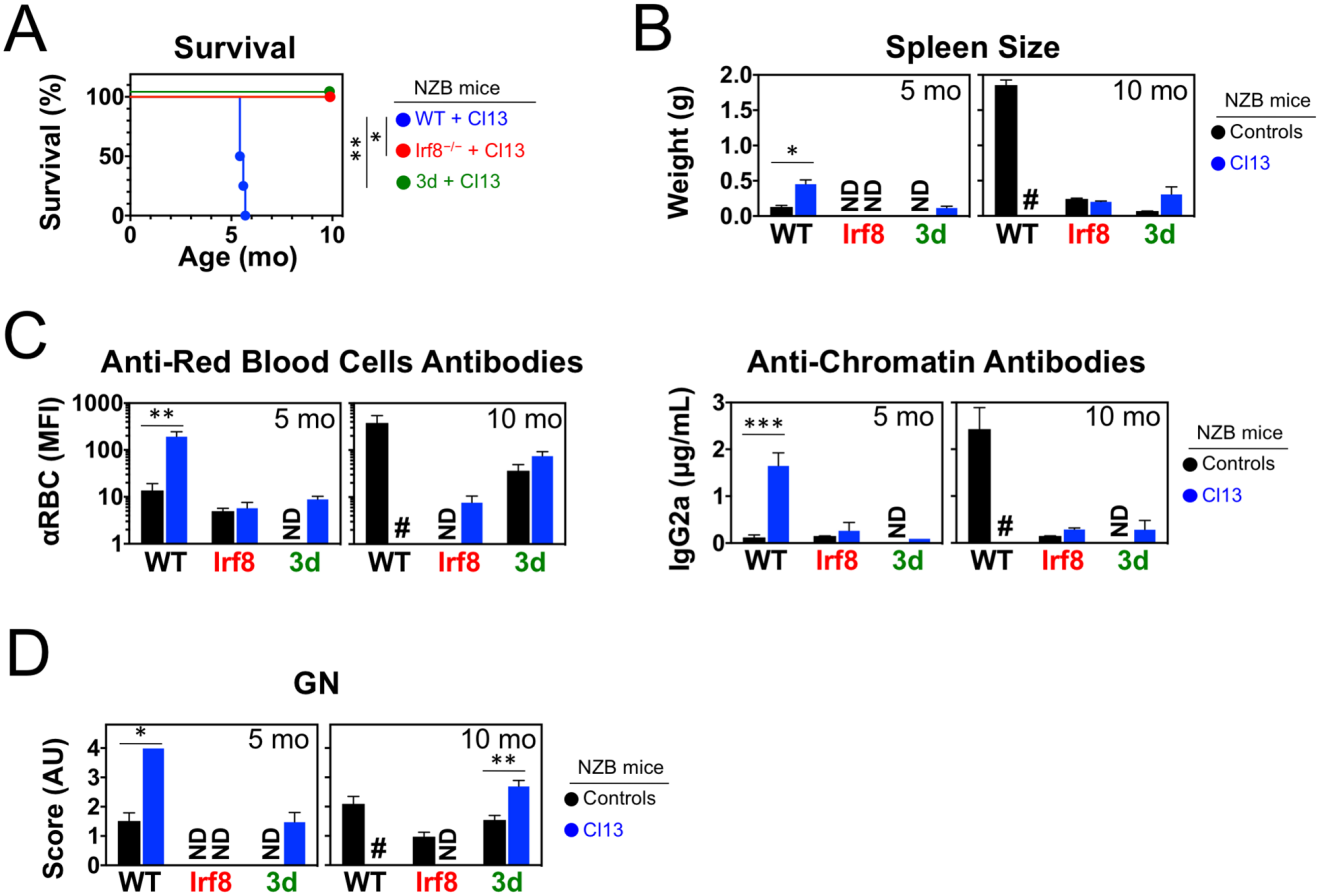
pDCs and endosomal TLRs are required for lupus development in NZB mice neonatally infected with LCMV. WT NZB mice, and NZB mice lacking either pDCs (*Irf8*^−/−^) or endosomal TLR signaling (*Unc93b1 3d* mutants), were infected <24 h after birth with LCMV Cl13 and analyzed at the indicated ages. Uninfected mice were used as controls. (**A**) Survival (*n* = 4–6 mice). (**B**) Spleen weight (*n* = 2–6 mice). (**C**) Anti-red blood cell and anti-chromatin autoantibodies (*n* = 4–10 mice). (**D**) Glomerulonephritis scores (*n* = 4–6 mice). Error bars (in B-C) indicate standard deviation and asterisks statistical significance (*, p<0.05; **, p<0.01; ***, p<0.001). ND, not determined.

The impaired endosomal TLR responses of pDCs from LCMV-infected NZB mice raised the possibility that these cells might not be critical for virus-induced disease acceleration. To address this issue, we assessed lupus progression in WT and *Irf8*^−/−^NZB mice neonatally infected or not with LCMV. As previously reported [30], *Irf8*^−/−^NZB mice lacked pDCs due to developmental arrest and were protected from spontaneous lupus-like disease, despite normal B cell responses to TLR stimulation and exogenous antigens. Although *Irf8*^−/−^NZB mice also lack CD8α^+^ DCs, these cells have not been implicated in systemic autoimmunity, and therefore reduced lupus-like disease in *Irf8*^−/−^NZB mice was attributed to lack of pDCs [30]. We found that persistent LCMV infection (0.89 ± 0.23 × 10^5^ PFU/mL at 3 mo of age) failed to precipitate lupus manifestations in pDC-deficient *Irf8*^−/−^NZB mice. Thus, while infected WT NZB mice showed 100% mortality at ~6 mo of age, all infected and uninfected *Irf8*^−/−^NZB mice were still alive when 10 mo-old (Fig 7A). Moreover, even at advanced age and regardless of whether they had been infected, IRF8-deficient mice showed no signs of disease, including splenomegaly (Fig 7B) and autoantibodies to erythrocytes or chromatin (Fig 7C). These results are consistent with the notion that pDCs are essential for virus-induced lupus disease acceleration in chronically infected mice.

### Sensing by endosomal TLRs mediates lupus enhancement in LCMV-infected NZB mice

Sensing of LCMV *via* TLR7 may be a major mechanism for disease enhancement in infected mice. However, LCMV also induces potent IFN-I and anti-viral T cell responses through TLR-independent pathways mediated by the cytosolic RNA sensor MDA5 (and possibly RIG-I) and signaling *via* the mitochondrial adaptor MAVS [45–51]. To determine whether virus-mediated innate immune activation by the MAVS pathway is of sufficient magnitude to sustain disease in the absence of endosomal TLR signaling, we examined the effect of LCMV infection in NZB mice congenic for the *3d* mutation of the *Unc93b1* gene, in which signaling by endosomal TLRs is extinguished and spontaneous disease averted [31]. In short-term experiments, LCMV-induced IFN-I production in NZB.*3d* mice was reduced at 16 h post-infection, but was comparable to WT NZB mice between 24 and 72 h post-infection (Panel A in S6 Fig). In contrast, this response was completely suppressed in homozygous double-deficient *3d/Mavs* mice of mixed NZB×C57BL/6 background lacking both endosomal TLR and MAVS signaling (Panel B in S6 Fig), confirming the contribution of the MAVS pathway to the LCMV response in *3d* mutant mice. However, despite the efficient MAVS-dependent innate immune response, NZB.*3d* mice were found to be protected from systemic autoimmunity even when persistently infected with LCMV. Thus, compared to infected WT NZB mice, infected NZB.*3d* mice with persistent LCMV infection (1.06 ± 0.18 × 10^5^ PFU/mL serum at 3 mo of age) survived significantly longer (Fig 7A) and, when 5 mo old, had normal spleen size (Fig 7B), undetectable levels of autoantibodies to red blood cells and chromatin (Fig 7C), and negligible kidney disease (Fig 7D). Similarly, at age 10 mo, infected NZB.*3d* mice showed no splenomegaly or anti-chromatin autoantibodies (Fig 7B and 7C), while GN scores were modestly increased compared to uninfected *3d* controls, suggesting weak TLR-independent effects on kidney pathology (Fig 7D). We conclude that, even though the *in vivo* anti-LCMV response is to a large extent mediated by cytosolic MAVS-dependent RNA sensors, endosomal TLR signaling is crucial for LCMV-induced disease acceleration in NZB mice.

## Discussion

In this study, we report that persistent infection with LCMV exacerbates systemic autoimmunity in lupus-prone NZB mice and even in female BXSB mice with mild spontaneous disease due to the absence of the Y chromosome-associated autoimmunity accelerator. Moreover, disease was abrogated in infected NZB mice lacking pDCs or endosomal TLR function, despite the evidence that LCMV strongly stimulates innate immune activation through the engagement of cytosolic RNA helicases and signaling *via* the adaptor MAVS in cDCs. These results are consistent with the critical role of endosomal nucleic acid sensing in systemic autoimmunity and demonstrate that potent TLR-independent IFN-I production by cells other than pDCs is insufficient for disease promotion.

Human studies to identify potential associations between viruses and autoimmunity are hampered by several limitations, including the possibility that specific infections may precipitate autoimmune disease in predisposed individuals but not in most people, and that disease-enhancing viruses may be cleared by the time symptoms of autoimmunity appear. Thus, as shown in this study, dissecting the interplay between virus infection and genetic susceptibility to autoimmunity and defining the mechanistic basis of disease modification may be facilitated by the use of well-characterized mouse and virus models, wherein host background genes, virus types, cell tropism, and persistence can be precisely controlled.

We found that chronic LCMV infection enhanced systemic autoimmunity in genetically predisposed but not in normal background mice. Association studies in SLE identified >80 genetic loci each contributing a small effect size, and thus aggregation of a sufficient number of them is required to attain the threshold for disease susceptibility [15]. However, in some individuals the number of predisposing gene variants is insufficient, and disease onset and severity depend on additional, most likely environmental, factors. Studies in mouse models of spontaneous lupus also showed disease dependency on multiple predisposing genes, with the contribution of few higher impact accelerator genes in some strains [52], such as the *Tlr7* gene duplication leading to increased TLR7 responses to RNA stimuli in male BXSB mice [35, 36]. Our result that LCMV infection converted the late, low-grade disease of female BXSB mice into an early disease almost as severe as the spontaneous disease of male BXSB mice indicates that certain infections may act as autoimmune disease modifiers by potentiating the effects of predisposing gene variants. In contrast, LCMV infection did not further enhance the severe disease of male BXSB mice, nor it induced autoimmune manifestations in non-susceptible C57BL/6 mice, indicating that the ability of a virus to trigger or worsen autoimmunity depends on the level of genetic predisposition. Thus, if the present results can be extended to human autoimmunity, one could envisage three categories of individuals in which disease might be differentially affected by superimposed viral infections: a) individuals with very low genetic predisposition, in which viral infections are insufficient to provoke autoimmunity; b) individuals with high genetic predisposition, who develop autoimmunity spontaneously without the need of an external stimulus; and c) individuals with limited genetic predisposition, which can however be complemented by infections that raise the risk level above a critical threshold. The low concordance of autoimmunity in identical twins suggests that most patients with autoimmune disease may fall in the latter group. Studying the combinatorial effects of infections and genetic susceptibility will therefore be essential to fully understand the pathogenesis of autoimmune diseases.

Several mechanisms have been evoked to explain how a virus may precipitate or accelerate autoimmunity, including molecular mimicry by viral antigens displaying sequence or conformational similarities to self-antigens, tissue damage leading to self-antigen release and presentation to previously quiescent or ignorant T and B cells, and bystander activation of autoreactive lymphocytes concurrent with the induction of the anti-viral response [19, 53]. In our model of LCMV-induced autoimmunity acceleration, molecular mimicry seems unlikely to be the main mechanism, given the relatively small LCMV genome (only encoding four proteins) as opposed to the diversity of B and T cell specificities in lupus-like disease. Tissue damage also appears not to be a main contributor, since LCMV is generally non-cytopathic, and virus-specific T cells that may cause immunopathology are deleted or tolerant in this model of neonatal infection [24, 34]. In contrast, although a conventional adaptive anti-viral response is not induced, virus-mediated activation of the innate immune system and the ensuing bystander activation of autoreactive B and T cells appeared critical factors for enhanced disease severity.

The requirement of pDCs and endosomal TLRs implicates viral nucleic acids, the main immunostimulatory feature of LCMV, in lupus acceleration. Nucleic acid recognition constitutes a key pathogenic mechanism of systemic autoimmunity [54, 55]. Self-nucleic acids in lupus can be presented in the form of autoantibody immune complexes, membrane-coated microparticles [56, 57], neutrophil extracellular traps (NETs) or oxidized mitochondrial DNA [58, 59], and can induce TLR7/9-dependent B cell activation and IFN-I production by pDCs and follicular DCs [2, 3, 60]. Although the mechanisms explaining accumulation of stimulatory nucleic acids in lupus have not been fully defined, excessive production and/or impaired elimination of nucleic acid-containing apoptotic materials have been implicated [61]. Excess of nucleic acids in SLE and other inflammatory/autoimmune conditions may also be due to rare deficiencies in specific nucleases [57, 62–66], modifications of nucleic acids that confer resistance to degradation [67], or aberrant expression of endogenous retroelements [68–70]. As shown in the present study, excess of stimulatory nucleic acids can also result from a persistent viral infection. In the case of LCMV, stimulatory RNA can occur within infectious or immature viral particles, as well as within exosome-like vesicles secreted by infected cells, shown to most effectively trigger TLR7-dependent IFN-I production by pDCs through a cell-cell contact-mediated mechanism [71]. Whether differences in nuclease activity or nucleic acid sensor signaling contribute to the differential effect of viral nucleic acids on autoimmunity in susceptible *vs*. resistant individuals remains to be determined.

In LCMV-infected NZB mice, pDCs were significantly expanded, activated, and strictly required for disease exacerbation. The non-redundant role of pDCs in this model is in line with the requirement of these cells for spontaneous disease. In lupus, pDCs can secrete large amounts of IFN-I particularly in response to nucleic acid-containing immune complexes, promote autoreactive B and T cell activation, accumulate in afflicted tissues, and produce chemokines that mediate inflammatory infiltrates [2, 72]. More directly, the key function of pDCs in lupus was shown by disease reduction in predisposed mice in which pDCs were absent, functionally impaired or transiently depleted [30, 73, 74], whereas lupus-like manifestations developed in Siglec-H-deficient mice with hyperresponsive pDCs [75]. Despite being required for lupus acceleration, pDCs from infected mice were hyporesponsive to endosomal TLR stimulation, consistent with the high frequency of LCMV^+^ pDCs and the evidence that the arenavirus-encoded nucleoprotein and Z protein exert inhibitory effects on IFN-I production in infected cells [76, 77]. Moreover, *in vivo* experiments showed that pDCs are not the main contributors to the innate response induced by LCMV, since pDC depletion reduced systemic IFN-I levels within the first 16–24 hours post-infection but had no effects on the peak IFN-I production at 24–72 hours post-infection [46, 50, 51]. Nonetheless, our results indicated that in LCMV-infected mice there is a sufficient number of newly emerging, as yet uninfected pDCs that serve crucial functions in the interplay of innate and adaptive autoimmune responses. In addition, it is conceivable that other innate cells contribute to virus-induced disease enhancement, particularly cDCs, the main producers of IFN-I in LCMV-infected mice [51], and follicular DCs, recently identified as a critical source of IFN-I in germinal centers in response to immune complexes of autoantibodies, ribonucleoproteins and complement, leading to autoreactive B cell activation [60].

As in spontaneous lupus, the engagement of endosomal TLRs was necessary for disease exacerbation in LCMV-infected NZB mice. Previous *in vivo* studies showed that LCMV potently induces IFN-I production even in the absence of endosomal TLRs, by activating the cytosolic RNA-sensing pathway mediated by MDA5 (and possibly RIG-I) and the adaptor MAVS [45–51]. The observation that high IFN-I production by this cytosolic pathway is not sufficient to elicit autoimmunity suggests that TLRs may be more efficient in the induction of other proinflammatory molecules, including IL-6 known to promote lupus in mice and humans [78]. In addition, endosomal TLRs may be required for their cell-intrinsic role in the activation of pDCs and other cell types following stimulation with nucleic acid-associated self-antigens and/or immune complexes, particularly B cells [31, 79, 80], cDCs [81], and follicular DCs [60]. Nevertheless, despite the central role of TLRs, hyperactivation of the cytosolic pathway by viral RNA may also contribute to disease acceleration. Consistent with this possibility, polymorphisms in the MDA5-encoding *IFIH1* gene have been associated with SLE and other autoimmune conditions [7–10], spontaneous or oxidative stress-induced aggregation of MAVS was observed in some SLE patients [11], and overexpression or gain-of-function mutation of MDA5 enhanced disease in lupus mice [12–14]. Additional studies with MAVS mutant and bone marrow chimeric mice will be required to elucidate the specific and combinatorial contributions of the endosomal and cytosolic pathways to spontaneous and virus-enhanced lupus. Nonetheless, our results suggest that the aggravating effect of an overactive cytosolic pathway in systemic autoimmunity is contingent on efficient endosomal TLR signaling.

We previously proposed that systemic autoimmunity may develop in a two-phase process [82]. The first phase is relatively inefficient and mediated by self-nucleic acids within apoptotic cell debris leading to a first wave of IFN-I production and autoreactive B and T cell activation, while the second phase involves a positive feedback loop mediated by nucleic acid-containing autoantibody immune complexes, which are taken up by pDCs and other cell types resulting in efficient TLR engagement and amplification of the inflammatory response. The present findings suggest that LCMV and possibly other viruses promote disease exacerbation primarily by accelerating the initiation phase of the autoimmune response. In a plausible scenario, owing to the expression of a diverse set of chemokine receptors and adhesion molecules [72], non-infected pDCs traffic to specific sites within lymphoid organs and inflamed tissues, where they encounter significant numbers of infected cells, particularly cDCs and macrophages. The uptake of exosome-associated viral RNA by pDCs [71] leads to TLR engagement and local production of large amounts of IFN-I followed by enhanced self-antigen presentation and activation of autoreactive B and T cells. Signaling *via* cytosolic RNA sensors and MAVS may further boost this early phase of the autoimmune process, although this pathway is ineffective in the absence of endosomal TLRs or pDCs. Viral nucleic acids may also promote the second phase, although, once induced, autoantibodies and related immune complexes are likely sufficient to sustain disease progression. Future studies in which LCMV clearance is induced at different time-points post-infection will clarify the effects of LCMV during the early *vs*. late phases of autoimmunity. Nonetheless, the importance of pDC activation at early disease stages is consistent with studies suggesting that pDC depletion [74] or IFNAR blockade [83] more efficiently reduce systemic autoimmunity when applied before a critical threshold of immune activation is exceeded.

Overall, using LCMV infection as a model, we showed that viral persistence caused severe disease exacerbation in lupus-prone but not in normal mice, demonstrating that viruses can act as triggers or amplifiers of autoimmune responses only if combined with critical predisposing abnormalities, including increases in self-antigen presentation, inflammatory stimuli, and autoreactive cells displaying heightened affinity and/or sensitivity to activation. Consistent with their primary role in spontaneous lupus, pDCs and the endosomal TLR pathway of innate immune activation were found to be necessary for disease acceleration in NZB mice, despite the efficient induction of IFN-I by viral RNA *via* cytosolic helicases and MAVS. Further experiments using additional virus and mouse models combined with well-designed clinical studies will be required to draw more definitive conclusions on the effects of virus infections on autoimmune disease expression and the underlying pathogenic mechanisms. Nevertheless, our current results suggest that therapeutic targeting of pDCs and endosomal TLRs may have universal applicability in SLE and other systemic autoimmune conditions, including when disease activity is magnified by a viral infection and/or an overactive cytosolic pathway of nucleic acid sensing.

## Supporting information

S1 Fig Neonatal LCMV infection has no effect on lupus-like disease in male BXSB mice or in male and female C57BL/6 mice. Mice were infected <24 h after birth with LCMV Cl13 and analyzed at the indicated ages. Uninfected mice were used as controls. (**A**) Anti-chromatin IgG2a autoantibodies detected by ELISA (sensitivity 5–10 ng/mL), proteinuria, and survival of LCMV-infected and control male BXSB mice (*n* = 5–9 mice). (**B**) Anti-chromatin IgG2a autoantibodies detected by ELISA (sensitivity 5–10 ng/mL), proteinuria, and survival of LCMV-infected and control C57BL/6 mice (*n* = 5–8 mice). No differences were noted between male and female C57BL/6 mice. Error bars indicate standard deviation, dots represent individual mice, and horizontal bars indicate average. All comparisons between infected and control mice showed no statistical differences.
(TIF)

S2 Fig Frequency of infected cells (LCMV^+^) in NZB mice neonatally challenged with LCMV Cl13. Infected were sacrificed at the age of 3 mo, and spleen cells were analyzed by FACS for the presence of intracellular LCMV using anti-NP antibodies (*n* = 3 mice). Non-infected mice were used as controls. Cell subsets analyzed included T1 B cells and ABCs (IgM^+^B220^+^ CD21^−^CD23^−^), follicular (FO) B cells (IgM^+^B220^+^ CD21^low^CD23^+^), marginal zone (MZ) B cells (IgM^+^B220^+^ CD21^+^CD23^−^), CD4^+^ and CD8^+^ T cells, cDCs (CD11c^+^ PDCA-1^−^), pDCs (CD11c^low^ PDCA-1^+^), macrophages (CD11c^−^ CD11b^low^ F4/80^+^), monocytes (CD11b^+^ CD11c^−^F4/80^−^) and monocyte subsets defined by Gr-1 marker expression. Numbers within FACS histograms indicate percentage of positive cells ± standard deviation.
(TIF)

S3 Fig Intrinsic and extrinsic effects of LCMV infection on the activation status of pDCs and cDCs from neonatally infected mice. NZB mice were infected with LCMV <24 h after birth and spleen cells analyzed at the age of 3 mo (*n* = 3 mice). (**A-D**) pDCs (CD11c^low^ PDCA-1^+^) and cDCs (CD11c^+^ PDCA-1^−^) from infected and control mice were analyzed for the expression of the activation markers CD86 and MHC class II (I-A^d^). pDCs and cDCs from infected mice were also analyzed after segregation into LCMV^+^ and LCMV^−^cells detected by intracellular staining using anti-LCMV-NP antibodies. Error bars indicate standard deviation, numbers within FACS histograms indicate percentage of positive cells ± standard deviation, and asterisks statistical significance (*, p<0.05; **, p<0.01; ***, p<0.001).
(TIF)

S4 Fig Effect of LCMV infection on the activation status of BM-derived pDCs. NZB mice were infected with LCMV <24 h after birth, and BM cells harvested at the age of 1.5 mo were differentiated into pDCs using Flt3L (*n* = 2 mice). (**A**) Efficiency of pDC (PDCA-1^+^B220^+^) differentiation. (**B**) Frequency of LCMV^+^ BM-derived pDCs detected by intracellular staining with anti-LCMV-NP antibodies. (**C-D**) BM-pDCs analyzed for the expression of the activation markers CD86 and MHC class I (H2-K^d^). BM-pDCs from infected mice were also analyzed after segregation into LCMV^+^ and LCMV^−^cells. Data are representative of 2 independent experiments. Error bars indicate standard deviation, numbers within FACS histograms indicate percentage of positive cells ± standard deviation, and asterisks statistical significance (*, p<0.05; **, p<0.01; ***, p<0.001).
(TIF)

S5 Fig *Irf8*^−/−^NZB mice lack pDCs and show impaired IFN-I production following *in vivo* CpG-mediated TLR9 stimulation. (**A**) Spleen cells from untreated WT and *Irf8*^−/−^NZB mice (2 mo old) were stained with antibodies to PDCA-1, CD11c and Siglec-H and analyzed by FACS. Shown are representative FACS plots of gated live spleen cells, where pDCs are identified as PDCA-1^+^CD11c^low^ or PDCA-1^+^Siglec-H^+^. (**B**) WT and *Irf8*^−/−^NZB mice (2 mo old) were injected with CpG ODN-2216 (TLR9 ligand), and serum levels of IFN-α levels were assessed by ELISA (sensitivity 10–20 pg/mL) 6 h post-injection (*n* = 4–5 mice). Data are representative of 3–4 independent experiments. Horizontal bars (in B) indicate average, and asterisks statistical significance (**, p<0.01).
(TIF)

S6 Fig NZB.*3d* mice lacking endosomal TLR signaling mount effective MAVS-dependent IFN-I response to LCMV. (**A-B**) NZB mice (WT), congenic *Unc93b1*^*3d/3d*^ mutant NZB mice lacking endosomal TLR signaling (3d), and *Unc93b1*^*3d/3d*^*Mavs*^*–/–*^mixed background (NZB×C57BL/6) mice lacking both endosomal TLR and MAVS signaling (3d/Mavs) were infected with LCMV (2 × 10^6^ PFU, i.v.) at the age of 2 mo (*n* = 3–4 mice). At the indicated time points post-infection, serum was analyzed for IFN-I levels using a sensitive ISRE-luc bioassay. Error bars indicate standard deviation, and asterisks statistical significance (*, p<0.05).
(TIF)
