# Supplementary figures and images for: Lupus acceleration by a MAVS-activating RNA virus requires endosomal TLR signaling and host genetic predisposition
Source: PLoS One. 2018 Sep 10;13(9):e0203118. doi: 10.1371/journal.pone.0203118 (PMC6130858; doi:10.1371/journal.pone.0203118)

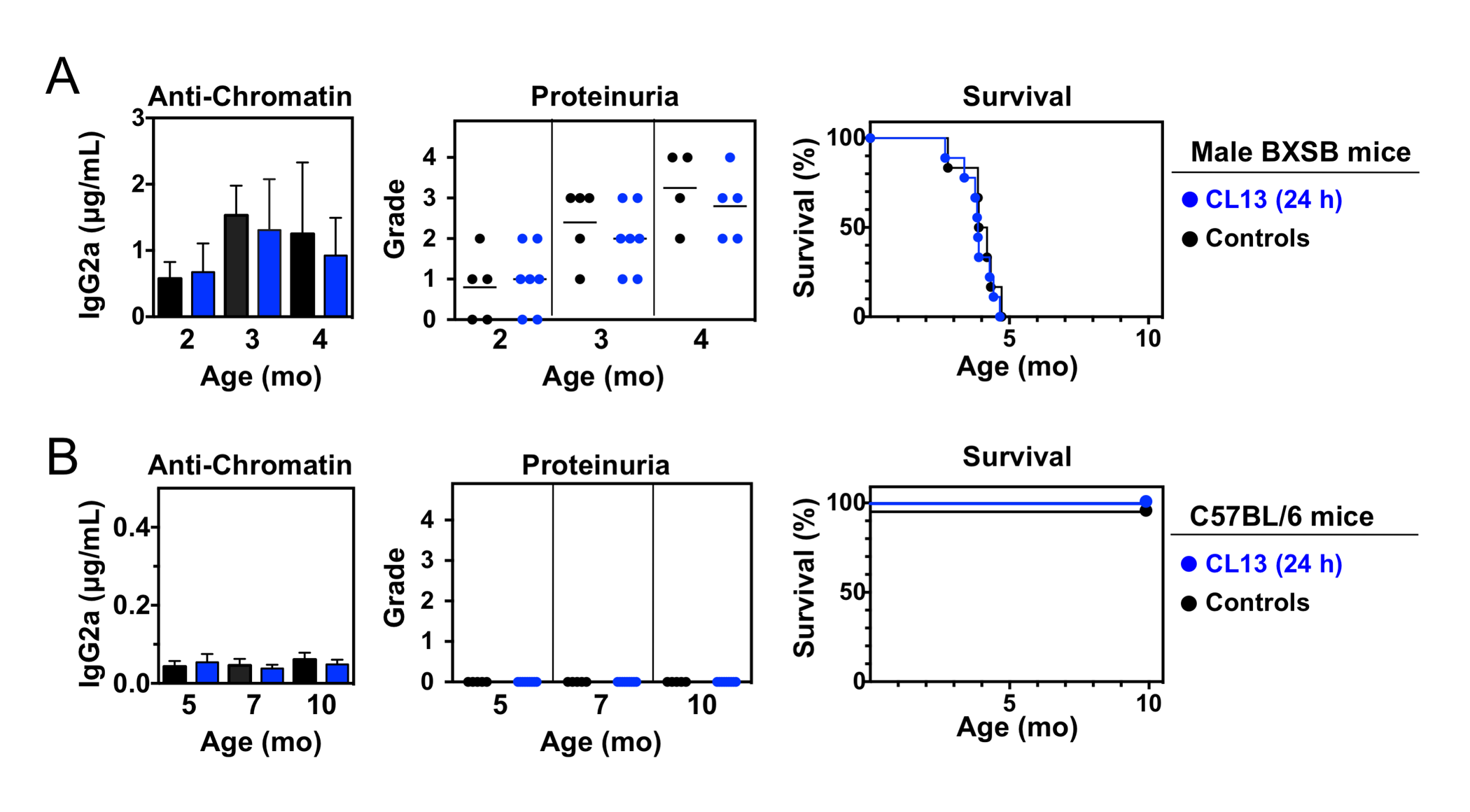

Supplement: S1 Fig — Mice were infected <24 h after birth with LCMV Cl13 and analyzed at the indicated ages. Uninfected mice were used as controls. (A) Anti-chromatin IgG2a autoantibodies detected by ELISA (sensitivity 5–10 ng/mL), proteinuria, and survival of LCMV-infected and control male BXSB mice (n = 5–9 mice). (B) Anti-chromatin IgG2a autoantibodies detected by ELISA (sensitivity 5–10 ng/mL), proteinuria, and survival of LCMV-infected and control C57BL/6 mice (n = 5–8 mice). No differences were noted between male and female C57BL/6 mice. Error bars indicate standard deviation, dots represent individual mice, and horizontal bars indicate average. All comparisons between infected and control mice showed no statistical differences. (TIF) [file pone.0203118.s001.tif]

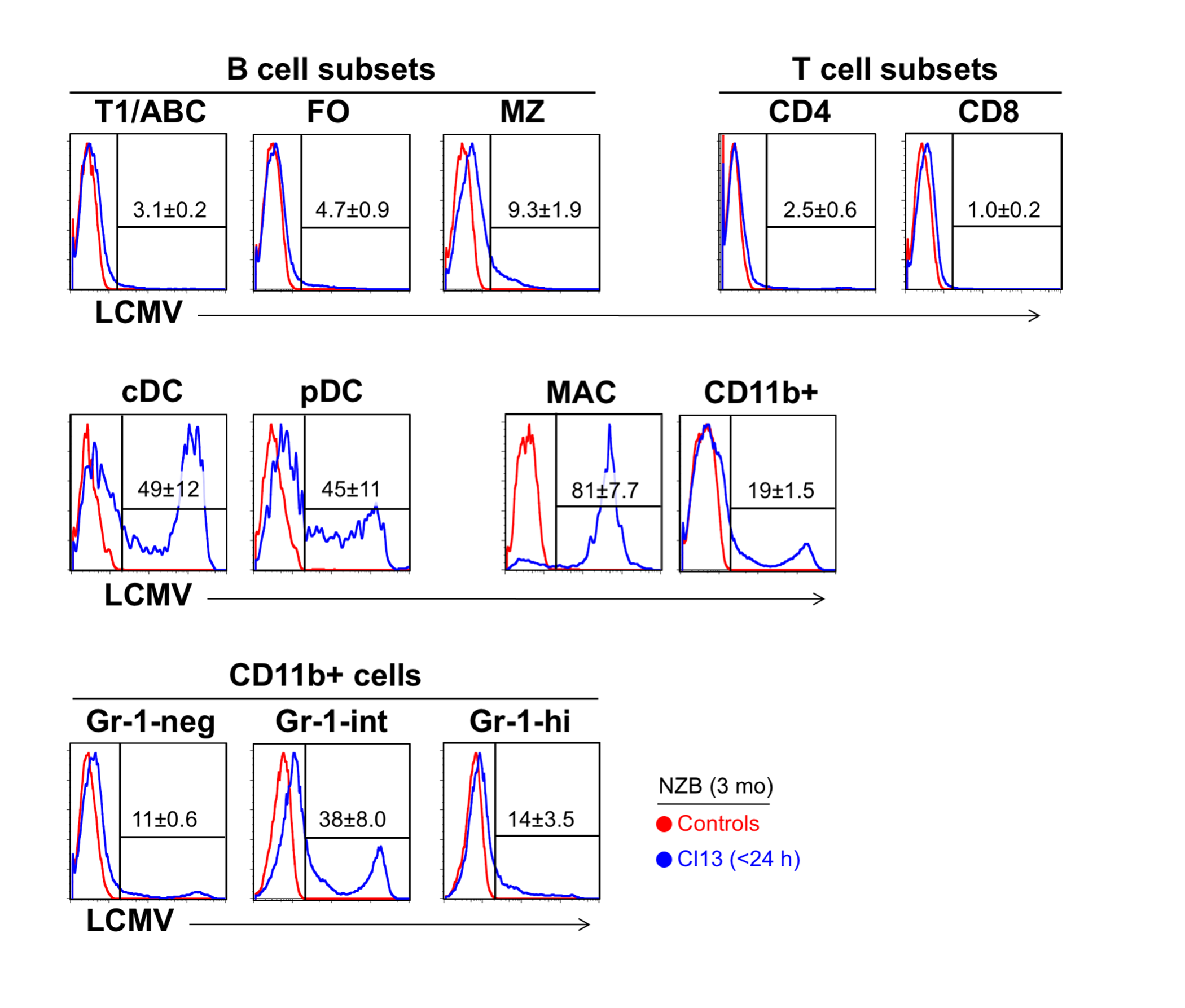

Supplement: S2 Fig — Infected were sacrificed at the age of 3 mo, and spleen cells were analyzed by FACS for the presence of intracellular LCMV using anti-NP antibodies (n = 3 mice). Non-infected mice were used as controls. Cell subsets analyzed included T1 B cells and ABCs (IgM+B220+ CD21−CD23−), follicular (FO) B cells (IgM+B220+ CD21lowCD23+), marginal zone (MZ) B cells (IgM+B220+ CD21+CD23−), CD4+ and CD8+ T cells, cDCs (CD11c+ PDCA-1−), pDCs (CD11clow PDCA-1+), macrophages (CD11c− CD11blow F4/80+), monocytes (CD11b+ CD11c−F4/80−) and monocyte subsets defined by Gr-1 marker expression. Numbers within FACS histograms indicate percentage of positive cells ± standard deviation. (TIF) [file pone.0203118.s002.tif]

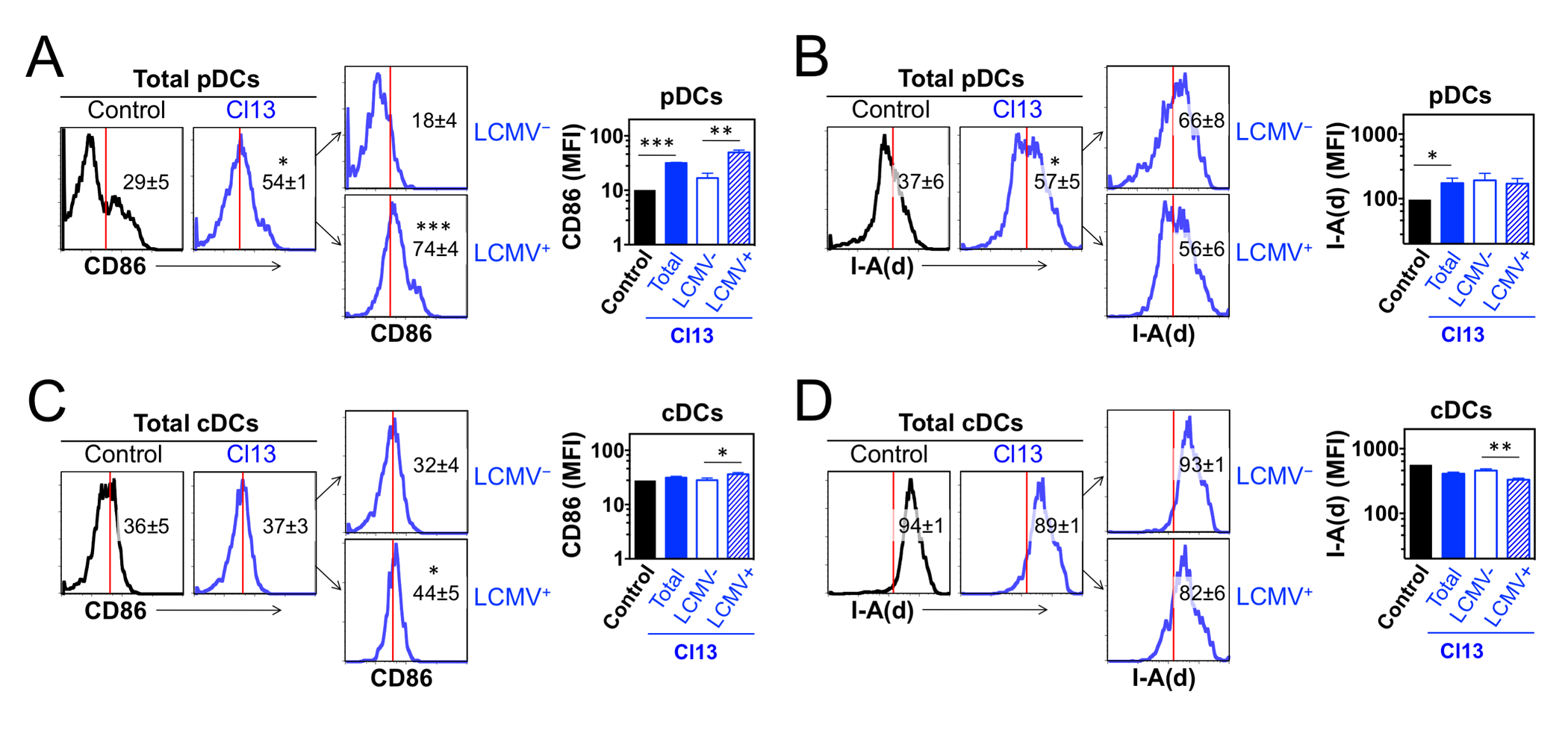

Supplement: S3 Fig — NZB mice were infected with LCMV <24 h after birth and spleen cells analyzed at the age of 3 mo (n = 3 mice). (A-D) pDCs (CD11clow PDCA-1+) and cDCs (CD11c+ PDCA-1−) from infected and control mice were analyzed for the expression of the activation markers CD86 and MHC class II (I-Ad). pDCs and cDCs from infected mice were also analyzed after segregation into LCMV+ and LCMV−cells detected by intracellular staining using anti-LCMV-NP antibodies. Error bars indicate standard deviation, numbers within FACS histograms indicate percentage of positive cells ± standard deviation, and asterisks statistical significance (*, p<0.05; **, p<0.01; ***, p<0.001). (TIF) [file pone.0203118.s003.tif]

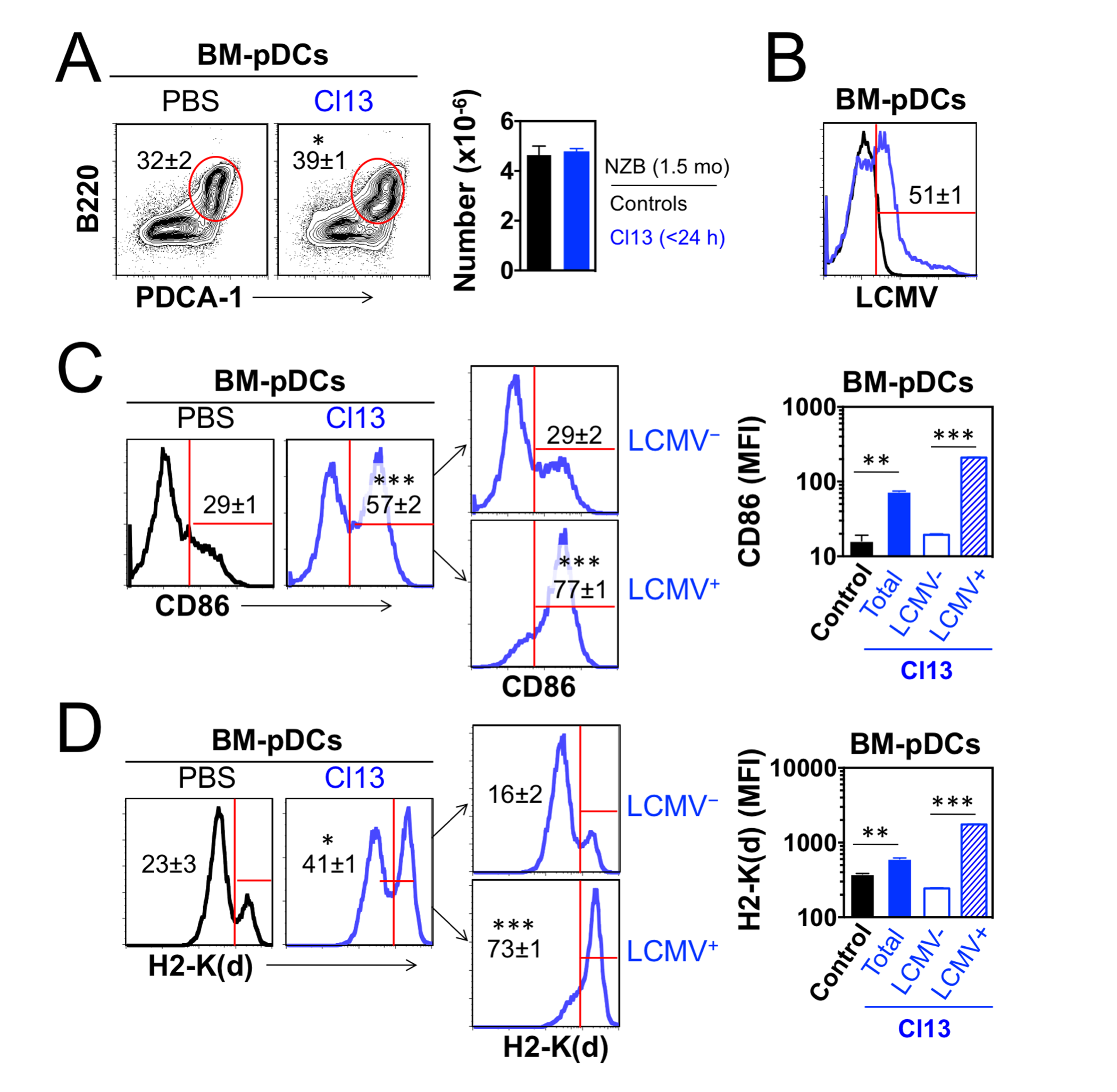

Supplement: S4 Fig — NZB mice were infected with LCMV <24 h after birth, and BM cells harvested at the age of 1.5 mo were differentiated into pDCs using Flt3L (n = 2 mice). (A) Efficiency of pDC (PDCA-1+B220+) differentiation. (B) Frequency of LCMV+ BM-derived pDCs detected by intracellular staining with anti-LCMV-NP antibodies. (C-D) BM-pDCs analyzed for the expression of the activation markers CD86 and MHC class I (H2-Kd). BM-pDCs from infected mice were also analyzed after segregation into LCMV+ and LCMV−cells. Data are representative of 2 independent experiments. Error bars indicate standard deviation, numbers within FACS histograms indicate percentage of positive cells ± standard deviation, and asterisks statistical significance (*, p<0.05; **, p<0.01; ***, p<0.001). (TIF) [file pone.0203118.s004.tif]

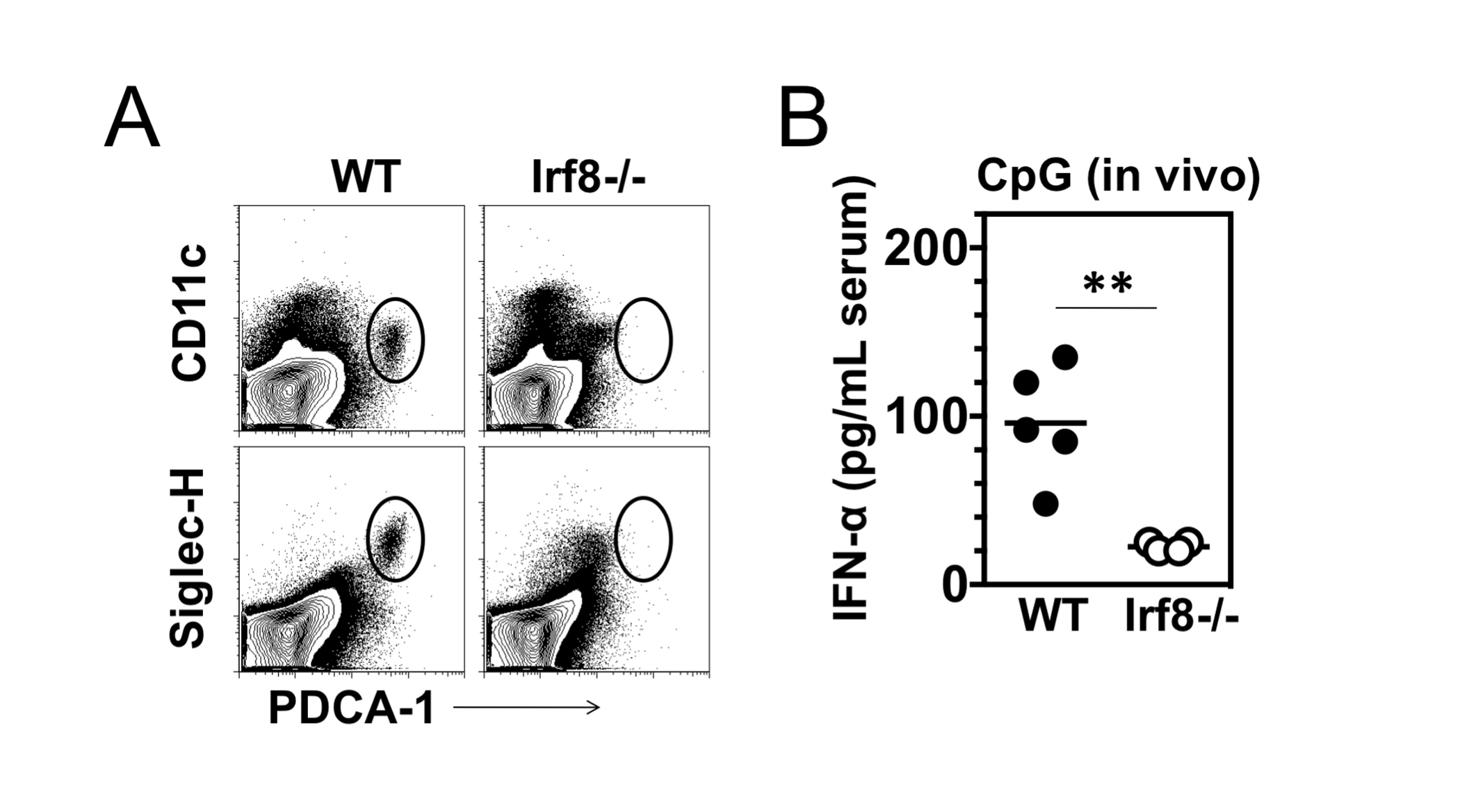

Supplement: S5 Fig — (A) Spleen cells from untreated WT and Irf8−/−NZB mice (2 mo old) were stained with antibodies to PDCA-1, CD11c and Siglec-H and analyzed by FACS. Shown are representative FACS plots of gated live spleen cells, where pDCs are identified as PDCA-1+CD11clow or PDCA-1+Siglec-H+. (B) WT and Irf8−/−NZB mice (2 mo old) were injected with CpG ODN-2216 (TLR9 ligand), and serum levels of IFN-α levels were assessed by ELISA (sensitivity 10–20 pg/mL) 6 h post-injection (n = 4–5 mice). Data are representative of 3–4 independent experiments. Horizontal bars (in B) indicate average, and asterisks statistical significance (**, p<0.01). (TIF) [file pone.0203118.s005.tif]

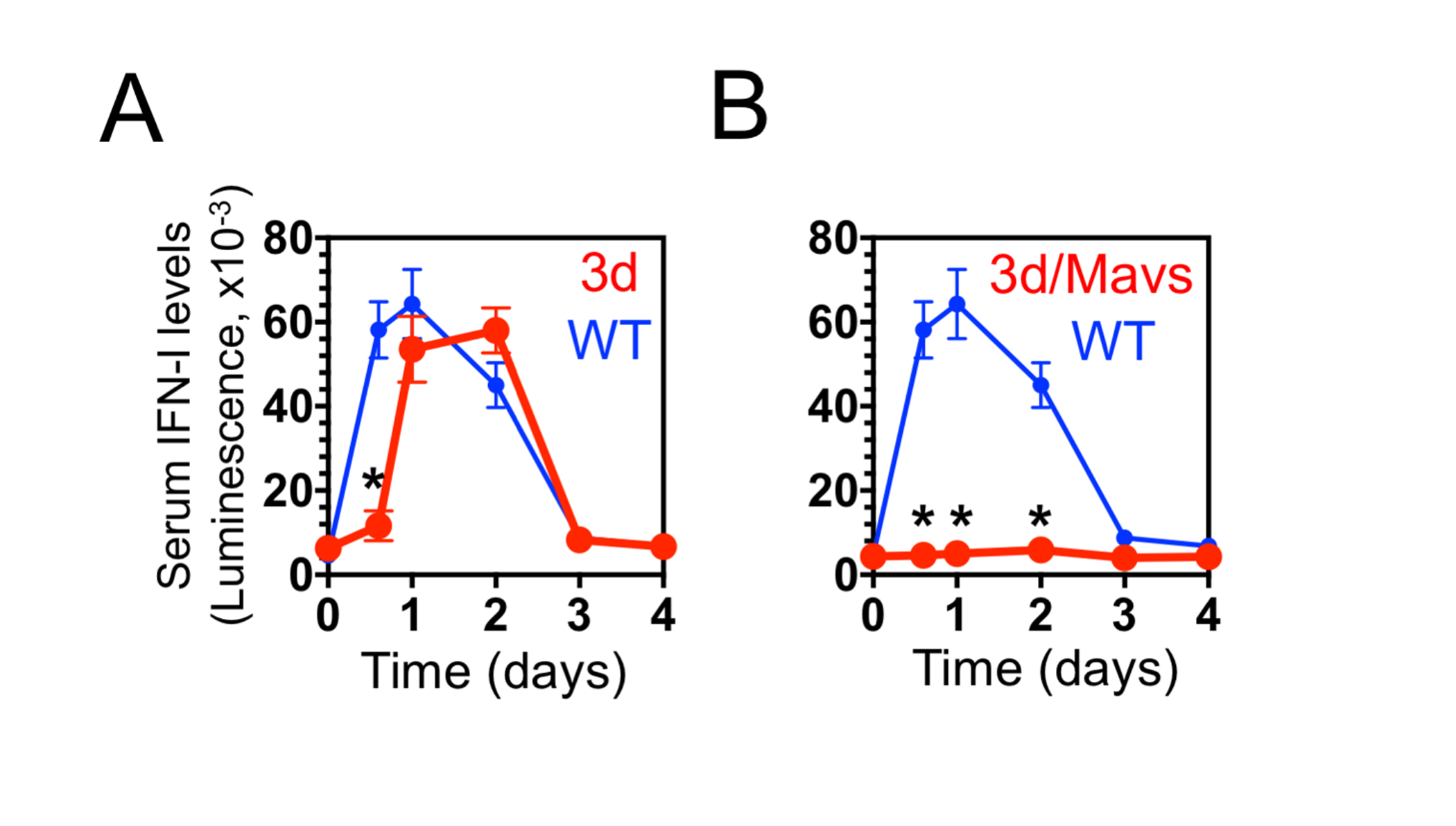

Supplement: S6 Fig — (A-B) NZB mice (WT), congenic Unc93b13d/3d mutant NZB mice lacking endosomal TLR signaling (3d), and Unc93b13d/3dMavs–/–mixed background (NZB×C57BL/6) mice lacking both endosomal TLR and MAVS signaling (3d/Mavs) were infected with LCMV (2 × 10^6^ PFU, i.v.) at the age of 2 mo (n = 3–4 mice). At the indicated time points post-infection, serum was analyzed for IFN-I levels using a sensitive ISRE-luc bioassay. Error bars indicate standard deviation, and asterisks statistical significance (*, p<0.05). (TIF) [file pone.0203118.s006.tif]
